# Examining the unique relationships between problematic use of the internet and impulsive and compulsive tendencies: network approach

**DOI:** 10.1192/bjo.2024.59

**Published:** 2024-05-09

**Authors:** Chang Liu, Kristian Rotaru, Lei Ren, Samuel R. Chamberlain, Erynn Christensen, Mary-Ellen Brierley, Karyn Richardson, Rico S. C. Lee, Rebecca Segrave, Jon E. Grant, Edouard Kayayan, Sam Hughes, Leonardo F. Fontenelle, Amelia Lowe, Chao Suo, René Freichel, Reinout W. Wiers, Murat Yücel, Lucy Albertella

**Affiliations:** BrainPark, Turner Institute for Brain and Mental Health, Monash University, Australia; BrainPark, Turner Institute for Brain and Mental Health, Monash University, Australia; and Monash Business School, Monash University, Australia; Military Medical Psychology Section, Logistics University of the People's Armed Police Force, Tianjin, China; and China and Military Mental Health Services and Research Centre, Tianjin, China; Department of Psychiatry, University of Southampton, UK; and Southern Gambling Clinic and Specialist Clinic for Impulsive/Compulsive Disorders, Southern Health NHS Foundation Trust, Southampton, UK; BrainPark, Turner Institute for Brain and Mental Health, Monash University, Australia; and Melbourne Centre for Behaviour Change, Melbourne School of Psychological Sciences, University of Melbourne, Australia; Department of Psychiatry and Behavioural Neuroscience, University of Chicago, USA; Department of Psychology, University of Amsterdam, the Netherlands; Melbourne School of Psychological Sciences, University of Melbourne, Australia; Obsessive, Compulsive, and Anxiety Spectrum Research Program, Institute of Psychiatry, Federal University of Rio de Janeiro, Rio de Janeiro, Brazil; and D'Or Institute for Research and Education, Rio de Janeiro, Brazil; Addiction Development and Psychopathology (ADAPT) Laboratory, Department of Psychology, and Centre for Urban Mental Health, University of Amsterdam, the Netherlands

**Keywords:** Compulsivity, impulsivity, network analysis, problematic use of the internet (PUI)

## Abstract

**Background:**

Both impulsivity and compulsivity have been identified as risk factors for problematic use of the internet (PUI). Yet little is known about the relationship between impulsivity, compulsivity and individual PUI symptoms, limiting a more precise understanding of mechanisms underlying PUI.

**Aims:**

The current study is the first to use network analysis to (a) examine the unique association among impulsivity, compulsivity and PUI symptoms, and (b) identify the most influential drivers in relation to the PUI symptom community.

**Method:**

We estimated a Gaussian graphical model consisting of five facets of impulsivity, compulsivity and individual PUI symptoms among 370 Australian adults (51.1% female, mean age = 29.8, s.d. = 11.1). Network structure and bridge expected influence were examined to elucidate differential associations among impulsivity, compulsivity and PUI symptoms, as well as identify influential nodes bridging impulsivity, compulsivity and PUI symptoms.

**Results:**

Results revealed that four facets of impulsivity (i.e. negative urgency, positive urgency, lack of premeditation and lack of perseverance) and compulsivity were related to different PUI symptoms. Further, compulsivity and negative urgency were the most influential nodes in relation to the PUI symptom community due to their highest bridge expected influence.

**Conclusions:**

The current findings delineate distinct relationships across impulsivity, compulsivity and PUI, which offer insights into potential mechanistic pathways and targets for future interventions in this space. To realise this potential, future studies are needed to replicate the identified network structure in different populations and determine the directionality of the relationships among impulsivity, compulsivity and PUI symptoms.

Problematic use of the internet (PUI), which involves excessive and/or otherwise problematic online behaviours, including excessive online gaming, social networking, shopping and pornography watching,^1^ poses a growing mental health research challenge due to its associated public health and societal costs.^1,2^ The weighted average prevalence of PUI is estimated to be 7.02% among the global population, often associated with decreased mental and physical health, impaired social functioning and productivity loss.^3^ Given the prevalence and potential negative consequences of PUI, it is important to understand its underlying mechanisms; this would allow for the development of targeted interventions.

Various theoretical frameworks have proposed that personality traits may play an important role in explaining individual differences in PUI. For instance, the Interaction of Person-Affect-Cognition-Execution (I-PACE) model states that certain personality traits (e.g. impulsivity) may predispose individuals to develop PUI.^4^ Meanwhile, compulsivity has been nominated as a primary construct for understanding transdiagnostic addictive behaviours (e.g. PUI) by expert consensus.^5^ Taken together, the European Cooperation in Science and Technology Action Programme proposed that both impulsivity and compulsivity should be considered as candidate constructs in understanding PUI.^2^

Impulsivity is broadly defined as the predisposition to act rashly when facing internal/external stimuli without thinking of consequences,^6^ and has been viewed as a hallmark feature of problematic behaviours, including PUI.^7^ As a multidimensional construct,^8^ impulsivity includes five interrelated facets, namely, negative urgency (the tendency to act rashly under strong negative emotions), positive urgency (the tendency to act rashly under strong positive emotions), lack of premeditation (the tendency to act without forethought), lack of perseverance (inability to stay focused on tasks) and sensation seeking (the tendency to seek novel, exciting experience). When these different facets of impulsivity were examined individually (as opposed to being merged into an overall impulsivity score), existing research found that these facets were not equally important to PUI.^9^ These findings demonstrated the internal heterogeneity of impulsivity, indicating that each facet of impulsivity should be examined separately in relation to PUI.

Compulsivity is defined as the tendency towards undertaking repetitive, habitual actions, whereby the original goal of the act has been lost.^10,11^ Core features of compulsivity include perfectionism, reward drive/cognitive rigidity and intolerance of uncertainty.^12^ Research on the association between compulsivity and PUI is still evolving. Several studies found that compulsivity is associated with increased PUI severity.^13^ It has been proposed that compulsivity may serve to maintain PUI via rigid coping responses when facing distress.^14^

While previous research has demonstrated that both impulsivity and compulsivity may be associated with PUI, there is limited understanding of how these constructs may be related to individual PUI symptoms. This drawback may be problematic in light of research showing that PUI may be composed of heterogeneous symptoms and that each of these symptoms may have unique relationships with risk factors.^15^ For instance, PUI symptoms characterised by interpersonal conflict (e.g. yelling when being bothered during internet use) may be particularly relevant to negative urgency, as high negative urgency may increase individuals’ propensity towards rash reactions when irritated. Thus, by looking specifically at the nuanced associations between risk-related traits and individual PUI symptoms, researchers may gain insights into the specific mechanisms that give risk to different PUI symptom profiles and inform more precise profile-targeted interventions for PUI.

One way of understanding how specific impulsive and compulsive traits may be related to individual PUI symptoms is through network analysis. As a graphic-based approach, network analysis enables researchers to estimate and visualise in an insightful way the complex interrelationships between predisposing variables and individual psychological symptoms.^16^ Within a network, impulsivity, compulsivity and PUI symptoms are depicted as nodes, which may directly connect to each other through edges between them.^17^ By inspecting the network structure, researchers may gain a direct understanding of which PUI symptoms are most closely related to a given predisposing variable and edges linking predisposing variables to individual PUI symptoms. Further, network analysis employs a concept known as ‘bridge centrality indices’ to statistically gauge the extent to which a specific node surpasses its originating psychological constructs and forms connections with theoretically independent constructs within the network.^18^^–20^ The bridge centrality index quantifies the extent to which a specific node within one subnetwork is connected to all other nodes in another subnetwork within the overarching network. This index is used to pinpoint nodes that are crucial for bridging different psychopathological constructs within the network. In the context of the current study, nodes with higher bridge centrality play a more pivotal role in connecting predisposing variables (e.g. impulsivity and compulsivity) and PUI.^18^

## Study aims

The current study represents the first application of network analysis to reveal the interrelations among impulsivity, compulsivity and individual PUI symptoms. By examining the network structure and bridge centrality, we aimed to (a) ascertain the specific edges among impulsivity, compulsivity and individual PUI symptoms, and (b) quantify the extent to which each predisposing variable is linked to the PUI symptom community (subnetwork) and identify the most influential bridge nodes in the network.

## Method

### Participants

The study engaged individuals who reside in Australia, recruited from two sources. The first group consisted of community members sourced through social media advertisement outreach, while the second comprised online users recruited via the Prolific crowdsourcing platform (www.prolific.com).

To be considered for this study, participants needed to be adults, 18 years or older, who had given their informed consent. Out of the eligible participants (*n* = 878), 397 completed measures assessing traits of impulsivity and compulsivity. However, from this subset, only 370 participants reported excessive internet use within the past three months (by responding ‘yes’ to the question ‘Have you used the internet excessively in the past three months?’) and, as a result, completed the PUI measure. Therefore, the present analyses incorporated data from these 370 individuals. The sample size exceeds the minimum sample size required for an 18-node network.^21^ Notably, 50.3% of these participants demonstrated PUI as determined by the established cut-off score (IAT-12 > 30).^22^

Participants from the community were offered the opportunity to enter a draw for one of 50 JB HiFi vouchers, each worth AU$100, as compensation upon completion of the study. Meanwhile, participants recruited through Prolific received an hourly reimbursement of £7.50. The authors assert that all procedures contributing to this work comply with the ethical standards of the relevant national and institutional committees on human experimentation and with the Helsinki Declaration of 1975, as revised in 2008. All procedures involving human subjects were approved by the Ethics Committee of Monash University (Project ID: 24401). Written informed consent was obtained from all subjects.

### Measures

#### Short UPPS-P impulsivity scale (S-UPPS-P^23^)

This instrument consists of 20 items designed to measure impulsivity. The scale is divided into five distinct subscales, namely negative urgency (example item: ‘When I feel bad, I will often do things I later regret in order to make myself feel better now’), positive urgency (example item: ‘I tend to lose control when I am in a great mood’), lack of premeditation (example item: ‘I like to stop and think things over before I do them’), lack of perseverance (example item: ‘I finish what I start’) and sensation seeking (sample item: ‘I quite enjoy taking risks’). Participants are asked to rate their agreement with each statement on a scale from ‘strongly agree’ (1) to ‘strongly disagree’ (4). Scores from negative urgency, sensation seeking and positive urgency subscales were reverse coded, and all five subscale scores were utilised in the data analysis. The internal consistency (McDonald's ω) of each subscale in the current study was as follows: negative urgency (0.76), positive urgency (0.82), lack of premeditation (0.76), lack of perseverance (0.63) and sensation seeking (0.72).

#### The Cambridge-Chicago compulsivity trait scale (CHIT^24^)

This 15-item self-report measure covers broad aspects of compulsivity, including perfectionism or need for completion, habitual behaviour, reward-seeking, desire for high standards and avoidance of difficult-to-control situations.^24,25^ In the version applied in this study, responses ranged from ‘strongly disagree’ (0) to ‘strongly agree’ (3). The total score was employed in the data analysis, and the scale demonstrated acceptable internal consistency in the current study (McDonald's ω = 0.71).

#### Young's internet addiction test (IAT), short version (IAT-12^22^)

This is a 12-item measure of PUI. Participants who had indicated excessive internet use over the past three months were invited to complete the IAT. An example item is ‘How often do you lose sleep due to being online late at night?’ Response options range from ‘never’ (1) to ‘very often’ (5). Individual item scores were used in the data analysis. The scale exhibited good internal consistency in the current study (McDonald's ω = 0.87).

### Data analysis

The network was estimated using the Gaussian graphical model (GGM), an undirected network where edges reflect partial correlations between nodes after controlling for all other nodes in the network. In our study, GGM was estimated based on Spearman's partial correlation, which calculates the pairwise relationships between nodes while adjusting for the effects of all other nodes within the network. We preferred Spearman's partial correlation over Pearson's, due to the former's resilience to skewed data, making it suitable for non-normally distributed data.^26^

We used R, version 3.3.3 for Mac OS (R Foundation for Statistical Computing) to perform the network analysis. For regularisation, we used the Extended Bayesian Information Criterion Graphical Least Absolute Shrinkage and Selection Operator (EBICglasso) procedure. This regularisation approach minimises trivial and minor coefficients to zero, reducing false-positive edges and generating a sparse network composed of the most robust edges.^27^ To strike a balance between sensitivity and specificity, we set the regularisation penalty term to 0.5.^27^ The Fruchterman-Reingold algorithm^28^ was utilised for network visualisation. Within these visualised networks, correlation magnitude was represented by edge thickness, with thicker edges indicating stronger correlations. Positive correlations were designated by blue edges and negative correlations by red edges, and nodes with stronger connections were situated closer together. The R package qgraph (version 1.9.2)^29^ was utilised for network estimation and visualisation.

The nodes in the displayed networks were pre-grouped into two communities, specifically the trait community (subscale scores of the S-UPPS-P scale and CHIT sum score) and the symptom community (individual items from the IAT scale). Bridge expected influence was employed to quantify how much each trait might connect to the PUI symptom community and identify influential bridge nodes. The concept of bridge expected influence tallies the total connectivity (i.e. sum of edge weights) from a specific node within one community to all nodes in a separate community,^18^ application of which is advised when the network encapsulates both positive and negative edges.^18^ Theoretically, nodes with high positive bridge expected influence values hold a higher probability of disseminating influence and prompting activation within the connected community.^18^

We ascertained edge accuracy by plotting the 95% CI (using 2000 bootstrap samples) of the edge weights and computed bootstrapped difference tests for edge weights. The Correlation-Stability coefficient was calculated to estimate the stability of the bridge expected influence centrality measure using a case-dropping bootstrap approach (with 2000 bootstrap samples). Bootstrapped difference tests for node bridge centrality were also calculated. The minimum acceptable Correlation-Stability-coefficient is 0.25, though preferably above 0.5.^21^ These procedures were carried out using the R package bootnet (version 1.5.3).^30^

## Results

Table 1 presents the descriptive statistics of the examined variables. The sample was composed of 370 participants (51.1% female) with an average age of 29.8 (s.d. = 11.1). The majority of the participants were currently employed (*n* = 303, 81.9%), and 236 (63.7%) reported that they had attained a bachelor's degree or higher. Additionally, 72 participants (19.5%) disclosed receiving income support payments from the government.

**Table 1:** Descriptive information of demographic and study variables

Variable	M (s.d.) /*N* (%)
Age (M [s.d.])	29.8 (11.1)
Female (*N* [%])	189 (51.1)
Employment status (*N* [%])	
Currently employed	303 (81.9)
Education level (*N* [%])	
Bachelor's degree or higher	236 (63.7)
Income Support recipient (*N* [%])	72 (19.5)
Negative urgency (M [s.d.])	10.2 (2.7)
Positive urgency (M [s.d.])	8.3 (2.7)
Lack of premeditation (M [s.d.])	7.4 (2.1)
Lack of perseverance (M [s.d.])	7.9 (1.9)
Sensation seeking (M [s.d.])	9.8 (2.8)
Compulsivity (M [s.d.])	27.9 (5.2)
IAT 1 (How often do you find that you stay online longer than you intended?) (M [s.d.])	4.1 (0.9)
IAT 2 (How often do you neglect household chores to spend more time online?) (M [s.d.])	3.2 (1.2)
IAT 3 (How often do your grades or schoolwork suffer because of the amount of time you spend online?) (M [s.d.])	2.6 (1.3)
IAT 4 (How often do you become defensive or secretive when anyone asks you what you do online?) (M [s.d.])	2.1 (1.2)
IAT 5 (How often do you snap, yell, or act annoyed if someone bothers you while you are online?) (M [s.d.])	1.9 (1.1)
IAT 6 (How often do you lose sleep due to late-night log-ins?) [M (s.d.)]	3.2 (1.3)
IAT 7 (How often do you feel preoccupied with the internet when offline, or fantasise about being online?) (M [s.d.])	2.4 (1.1)
IAT 8 (How often do you find yourself saying ‘Just a few more minutes’ when online?) (M [s.d.])	3.3 (1.3)
IAT 9 (How often do you try to cut down the amount of time you spend online and fail?) (M [s.d.])	2.7 (1.2)
IAT 10 (How often do you try to hide how long you have been online?) (M [s.d.])	1.9 (1.2)
IAT 11 (How often do you choose to spend more time online over going out with others?) (M [s.d.])	2.5 (1.4)
IAT 12 (How often do you feel depressed, moody, or nervous when you are offline, which goes away once you are back online?) (M [s.d.])	1.8 (1.1)
Total score > 30 (*N* [%])	186 (50.3)

M, mean; IAT, internet addiction test.

### Network estimation

**Fig. 1:**
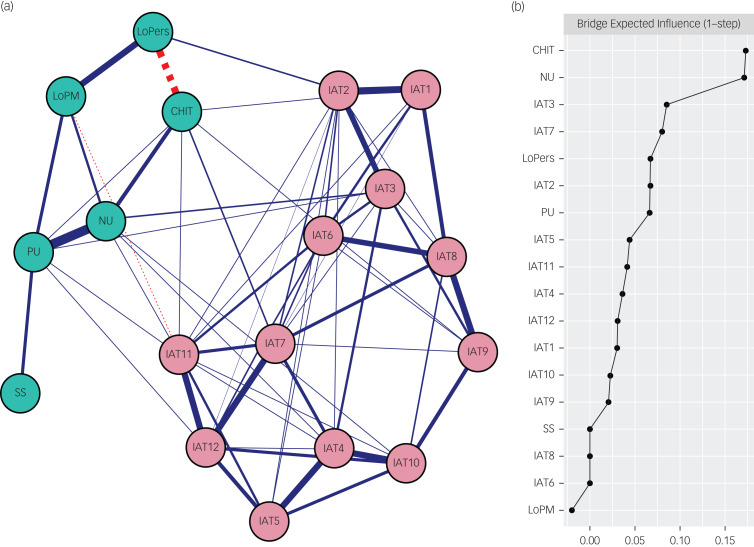
(a) Network structure of the estimated network. Solid edges represent positive correlations and dotted edges represent negative correlations. The thickness of the edge reflects the magnitude of the correlation. Cut value = 0.03. The text of problematic use of the internet symptoms can be seen in Table 1. (b) Bridge centrality plot. CHIT, compulsivity; NU, negative urgency; IAT, internet addiction test; LoPM, lack of premeditation; LoPers, lack of perseverance; SS, sensation seeking; PU, positive urgency.

The estimated network is depicted in Fig. 1(a). CHIT demonstrated a positive correlation with four PUI symptoms (IAT 2, IAT 6, IAT 7, IAT 11), with weights ranging from 0.02 to 0.08. The strongest edge emerged between compulsivity and IAT 7 (‘How often do you feel preoccupied with the internet when offline or fantasise about being online?’), yielding an edge weight of 0.08. Similarly, negative urgency was positively correlated with four PUI symptoms (IAT 3, IAT 4, IAT 5, IAT 10), with weights varying between 0.02 and 0.07. The strongest edge materialised between negative urgency and IAT 3 (‘How often do your grades or schoolwork suffer because of the amount of time you spend online?’), presenting an edge weight of 0.07. Positive urgency showed a positive correlation with four PUI symptoms (IAT 3, IAT 4, IAT 11, IAT 12) with weight ranging from <0.01 to 0.03. The most significant connection was observed between positive urgency and IAT 12 (‘How often do you feel depressed, moody or nervous when you are offline, which goes away once you are back online?’), giving an edge weight of 0.03. Lack of premeditation revealed a negative correlation with one PUI symptom, IAT 11 (‘How often do you choose to spend more time online over going out with others?’), presenting an edge weight of -0.02. Lack of perseverance was positively correlated with one PUI symptom, IAT 2 (‘How often do you neglect household chores to spend more time online?’), giving an edge weight of 0.07. Sensation seeking exhibited no association with any PUI symptoms. Bootstrapped CIs of each node (Supplementary Figure 1 available at https://doi.org/10.1192/bjo.2024.59) and bootstrapped edge weight difference test (Supplementary Figure 2) are provided in the Supplementary Materials.

### Bridge centrality

Raw bridge expected influence values are illustrated in Fig. 1(b). Two nodes displaying the highest bridge expected influence were identified – CHIT and negative urgency, followed by lack of perseverance, positive urgency, sensation seeking and lack of premeditation (in descending order of bridge centrality). The Correlation-Stability-coefficient for bridge expected influence is 0.28, surpassing the recommended cut-off value (i.e. 0.25). Results from bootstrapped stability tests (Supplementary Figure 3) and bootstrapped difference tests (Supplementary Figure 4) are presented in the Supplementary Materials.

## Discussion

This investigation stands as the first to scrutinise the unique relationships among impulsivity, compulsivity and PUI symptoms. One significant advancement facilitated by the current study lies in exposing the distinct relationships between well established predisposing factors (i.e. impulsivity and compulsivity) and PUI, while controlling for shared variances. Regarding our first aim, we discerned several distinct relationships among impulsivity traits, trait compulsivity and PUI symptoms (e.g. negative urgency-interpersonal conflict and positive urgency-withdrawal), with the sole negative relation appearing between lack of premeditation and neglect of social activities. Regarding our second aim, we discovered that trait compulsivity and negative urgency were the most influential bridge nodes in the network, thus affirming our hypothesis.

The European Cooperation in Science and Technology Action Programme called for research into elucidating the potential role of compulsivity in PUI.^2^ In response to this call, we investigated how trait compulsivity might uniquely relate to individual PUI symptoms. We found that trait compulsivity was closely tied to PUI symptoms characterised by negative consequences (e.g. sleep loss, neglect of household chores and neglect of social activities). This can be attributed to cognitive inflexibility, a hallmark of compulsivity.^31^ Specifically, inflexible individuals are more likely to struggle with adjusting their behavioural patterns,^32^ and hence are more likely to persistently engage in internet use despite experiencing aversive consequences such as sleep loss and failure to fulfil role obligations at home.

By pinpointing specific trait-symptom relationships, our results contribute to the ongoing debate over whether positive urgency and negative urgency should be considered as two distinct constructs (e.g. ^33,34^). Cyders et al^34^ argued that positive urgency is distinct from negative urgency as it explains unique variance in problematic behaviours that is not explained by negative urgency. Conversely, a meta-analysis contended that both traits demonstrated a relatively similar pattern of correlations across different mental disorders including substance-related addictions.^35^ Nevertheless, most empirical studies examining the roles of positive and negative urgency in psychopathology were based on the sum-score approach, which considered mental disorders as unitary constructs (indexed by symptom sum scores). As previously mentioned, this approach might conceal symptom heterogeneity and might potentially overlook different association patterns between predisposing variables and symptoms. Supporting this viewpoint, we found some unique relationships that might distinguish positive from negative urgency. For instance, positive urgency has a strong positive relationship with withdrawal (IAT 12), which is not observable for negative urgency. Moreover, no association was found between positive urgency and interpersonal conflict (IAT 5), which is pronounced for negative urgency only. These results suggest that, when controlling for the shared variance, positive and negative urgency differ in their co-occurring symptoms and support the notion that positive urgency and negative urgency may be considered as two distinct constructs.

Our results also help clarify the role of lack of perseverance in PUI. We found that lack of perseverance was uniquely related to neglect of household chores (IAT 2). One theory posits that the association between lack of perseverance and PUI may be explained by intrusive thoughts in relation to the internet, as such thoughts may trigger craving, leading to excessive internet use.^9^ However, we did not find any association between lack of perseverance and fantasising about being online (IAT 7). The unique association between lack of perseverance and neglect of household chores may suggest a procrastinatory use of the internet,^36,37^ with individuals high on lack of perseverance using the internet to procrastinate about intended but dull tasks (e.g. doing household chores).

Interestingly, we found a distinct negative relationship between lack of premeditation and neglect of social activities (IAT 11). A possible explanation for this association may be that people characterised by lack of premeditation tend to be less organised and may rush into things without forethought. Thus, instead of purposefully choosing between spending more time online and going out with others, these individuals may randomly allocate their time to either of these activities.

Our study aligns with earlier research,^9,38^ failing to find connections between sensation seeking and PUI. This may be attributed to IAT 12 focusing solely on addictive PUI. Sensation seeking could be more applicable to dangerous and antisocial PUI types, not addictive PUI.^39,40^ Future research should explore differences in network connectivity between sensation seeking and various internet usage types.

Our network's node bridge centrality offers insights into the relative importance of impulsivity and compulsivity in connection to the PUI symptom community. As hypothesised, compulsivity and negative urgency emerged as bridge nodes within the network. The significant role of negative urgency aligns with previous research involving Chinese university students that found negative urgency to have the most significant impact on PUI (among the five UPPS-P facets).^38^ Crucially, our results underscore the primary role of compulsivity in PUI, indicating it may characterise a behavioural phenotype of PUI.

In theory, addressing nodes with high bridge centrality could deactivate the symptom community. Both compulsivity and negative urgency might be associated with impaired cognitive functioning (i.e. cognitive flexibility and inhibitory control). Consequently, cognitive training focusing on flexibility and inhibitory control may effectively reduce these traits. Further, digital personality change interventions have shown promising results in reducing unwanted traits (e.g. neuroticism).^41^ Future research should examine the applicability of such interventions in reducing compulsivity and negative urgency.

Despite the promise of our findings, there are several limitations that merit consideration. First, given the cross-sectional design, causal relationships cannot be definitively established among the studied variables. Future research should strive to confirm these findings with longitudinal data. Second, the variables in this study were examined through self-report measures, inducing potential reporting errors and shared method variance. However, these measures capture in a concise and convenient manner a wealth of information about traits. Third, the current results were generated from a community sample; thus, there are limitations regarding the extent to which these findings would apply in the clinical world. Future studies should aim to replicate our findings in clinical contexts, such as with individuals exhibiting severe levels of PUI or those currently undergoing PUI treatment. Fourth, despite our study meeting the minimum sample size requirement (153 individuals for an 18-node network),^21^ the network stability was acceptable but not optimal. It would be beneficial if future studies attempted to replicate current findings under conditions of optimal stability. Last, bridge nodes theoretically have the potential to activate the symptom community,^18^ and this assumption needs to be empirically tested.

### Future directions

In our study, we recognised compulsivity as one of the influential nodes in relation to the PUI symptom community. Despite its multidimensional nature, there is no consensus on the specific dimensions included in the compulsivity constructs. Future research should aim to (a) determine compulsivity's constituent dimensions, and (b) examine the relationships between different compulsivity dimensions and PUI symptoms. This information may help identify the critical compulsivity dimension related to PUI symptoms, informing more precise prevention and interventions.

The current network was estimated on cross-sectional, between-subject data. Given the mixed evidence on the validity of using centrality metrics derived from cross-sectional data to predict symptom changes over time, and concerns over whether results from between-subject data may predict personalised dynamic processes,^42^ it is crucial for future studies to evaluate our findings using time series data with dynamical systems approaches.

## Conclusion

Our study is the first exploratory endeavour to apply network analysis to model the intricate relationships between impulsivity, compulsivity and PUI symptomatology. Our findings began to illuminate the specific and distinct relationships between impulsivity, compulsivity and individual PUI symptoms, and pinpointed negative urgency and compulsivity as influential bridging nodes. To enhance the robustness and applicability of our findings, it is essential to verify the identified network structure in independent data-sets and determine the directions of relationships using longitudinal data. Conducting these replication and extension studies across both subclinical and clinical populations will establish a solid base for translating the findings into prevention and intervention strategies.

## Supporting information

Liu et al. supplementary material Liu et al. supplementary material

## Data Availability

The data that support the findings of this study are available from the corresponding author, C.L., on reasonable request.
