# Supplementary material for: Examining the unique relationships between problematic use of the internet and impulsive and compulsive tendencies: network approach
Source: BJPsych Open. 2024 May 9;10(3):e104. doi: 10.1192/bjo.2024.59 (PMC11094446; doi:10.1192/bjo.2024.59)
Supplement: Liu et al. supplementary material [file S2056472424000590sup001.docx]

**Supplementary Materials**

**The results of impulsivity/compulsivity-PUI network**

1. Figure S1. Accuracy of edge weights
2. Figure S2. Bootstrapped difference test for edge weights
3. Figure S3. Stability of node bridge expected influence
4. Figure S4. Bootstrapped difference test for node bridge expected influence


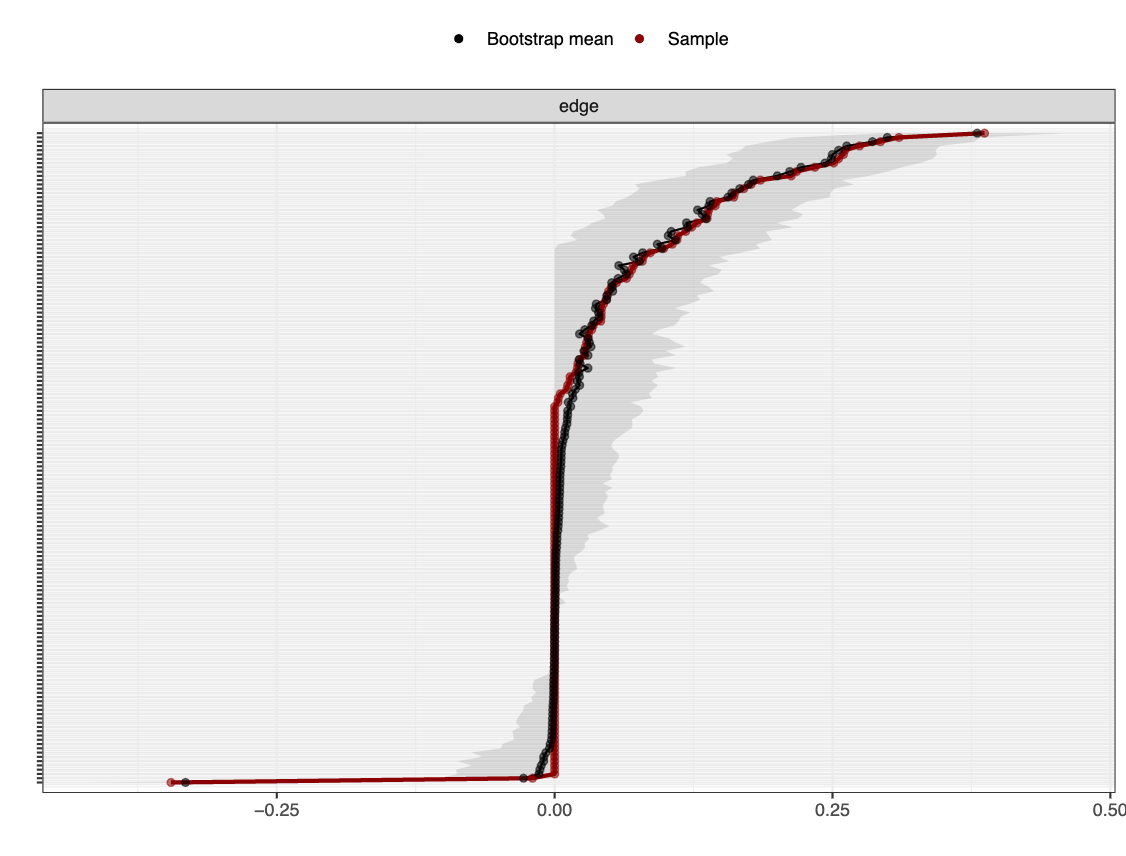


Figure S1. Accuracy of edge weights

*Note*: The red line depicts the sample edge weights and the gray bar depicts the bootstrapped confidence interval.


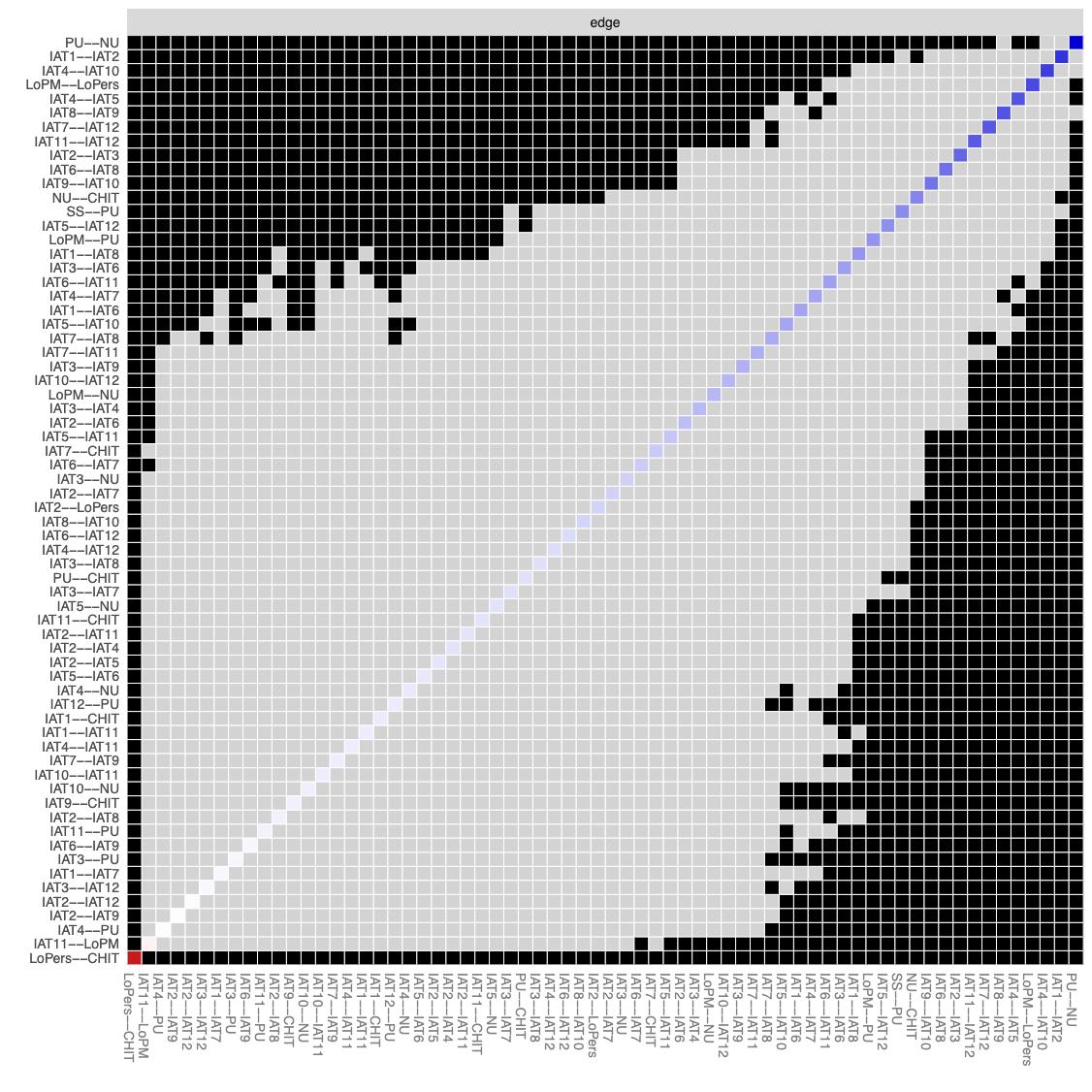


Figure S2. Bootstrapped difference test for edge weights

*Note*: Gray boxes indicate edge weights that do not differ significantly from one another, while black boxes indicate edge weights that do differ significantly. Blue and red boxes on the diagonal correspond to edge weights with positive and negative correlations, respectively.

Figure S3. Stability of node bridge expected influence

*Note*: The red bar represents the average correlation between node bridge expected influences in the full sample and subsample with the red area depicting the 2.5th quantile to the 97.5th quantile.


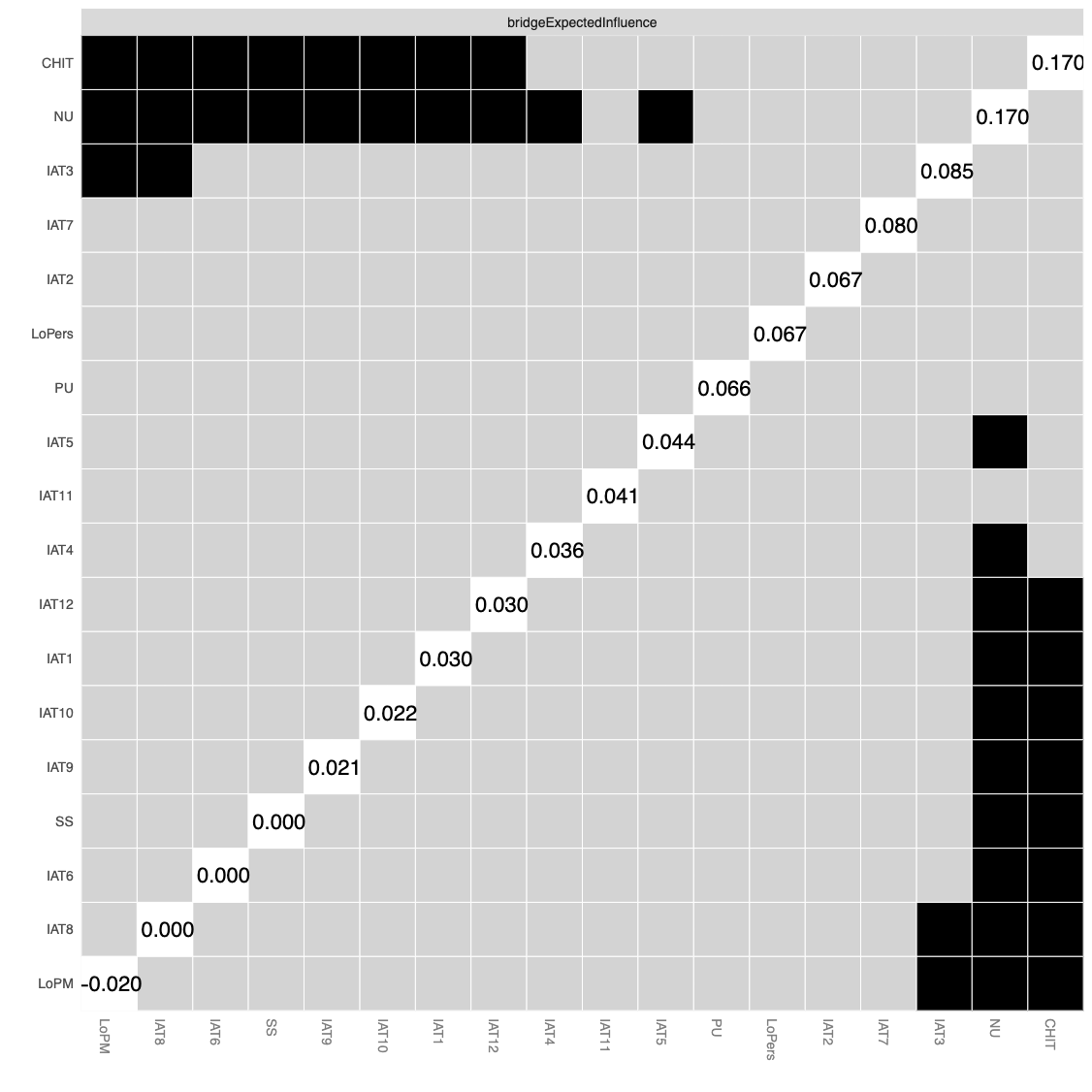


Figure S4. Bootstrapped difference test for node bridge expected influence

*Note*: Gray boxes indicate node bridge expected influences that do not differ significantly from one another, while black boxes indicate node bridge expected influences that do differ significantly.
